# Supplementary material for: Chinese sign language recognition based on surface electromyography and motion information
Source: PLoS One. 2023 Dec 7;18(12):e0295398. doi: 10.1371/journal.pone.0295398 (PMC10703204; doi:10.1371/journal.pone.0295398)
Supplement: Attachment — Submitted filename: Response to Reviewers.pdf [file pone.0295398.s002.pdf]

Dear Reviewers,

I hope this email finds you well. I would like to express my sincere appreciation for your time and effort in reviewing my manuscript, submitted for publication in PLOS One. I have carefully considered the comments and suggestions provided by the reviewers, and I am grateful for their valuable input.

I am pleased to submit a revised version of the manuscript, addressing the reviewers' comments and incorporating necessary revisions. Below, I have outlined the major comments from the reviewers and detailed how I have addressed each of them in the revised manuscript:

Reviewer 1:

1. I have inserted some references related to sEMG signals for pattern recognition in complex tasks in the manuscript.

2. The relevant information on sEMG, sampling frequency of acceleration and duration of gesture actions has been added to the manuscript.

3. During the revision, it was found that the calculation of gesture types was not related to text research, and the calculation of gesture types has been removed from the manuscript.

4. The CSL vocabulary is extensive.To facilitate the analysis, this study selects 120 words involving nine gestures and eight trajectories. These 120 words were selected based on usage rates from the "Chinese Sign Language".

5. The abbreviations VCM, FFE, and ET have been explained in Figures 8 and 9, and explanations have been added to the article

6. MAV is used to determine whether a word is a dynamic gesture vocabulary, for word segmentation, and is calculated based on the entire signal related to the word. It processes the sEMG and ACC signals of a gesture action.

7. The values of K and T have been added to the manuscript.

8. The threshold value is obtained by synthesizing the signal activity start and stop situations of 8 experimental volunteers during sign language action training.

9. The contain in line 210 has been deleted.

10. THGV is a writing error and has been changed to DHGV.

11. Added a discussion section and added relevant references.

Reviewer 2:

The first part of the results has been placed in the Materials and methods and a discussion section has been added.

In addition to addressing the specific comments, I have also made some minor revisions to improve the overall clarity and flow of the manuscript. A detailed summary of all the changes made can be found in the tracked changes version of the manuscript.

I believe that these revisions have significantly improved the quality of the manuscript and have addressed the concerns raised by the reviewers.

Thank you once again for the valuable feedback provided by the reviewers on my manuscript. I look forward to your reply Please do not hesitate to contact me if you require any further information or have any additional questions.

Sincerely,

Wenyu Li
