# Supplementary material for: Chinese sign language recognition based on surface electromyography and motion information
Source: PLoS One. 2023 Dec 7;18(12):e0295398. doi: 10.1371/journal.pone.0295398 (PMC10703204; doi:10.1371/journal.pone.0295398)
Supplement: Attachment — Submitted filename: Response to Reviewers.pdf [file pone.0295398.s003.pdf]

Dear Reviewers,

I hope this email finds you well. I would like to express my sincere appreciation for your time and effort in reviewing my manuscript, submitted for publication in PLOS One. I have carefully considered the comments and suggestions provided by the reviewers, and I am grateful for their valuable input.

I am pleased to submit a revised version of the manuscript, addressing the reviewers' comments and incorporating necessary revisions. Below, I have outlined the major comments from the reviewers and detailed how I have addressed each of them in the revised manuscript:

Reviewer 1:

Reviewer 2:

1.The performance comparison section has been added before the discussion.

2. Enriched the discussion of the results, in the discussion section.

3.A dot has been added.

4. The ethical approval and consent of the participants has been added at the participants.

5. The major contribution of the research has been added at the end of the introduction.

In addition to addressing the specific comments, I have also made some minor revisions to improve the overall clarity and flow of the manuscript. A detailed summary of all the changes made can be found in the tracked changes version of the manuscript.

I believe that these revisions have significantly improved the quality of the manuscript and have addressed the concerns raised by the reviewers.

Thank you once again for the valuable feedback provided by the reviewers on my manuscript. I look forward to your reply Please do not hesitate to contact me if you require any further information or have any additional questions.

Sincerely,

Wenyu Li
